# Blood biomarkers as surrogate endpoints in Alzheimer’s disease research

**DOI:** 10.3389/fnagi.2025.1576373

**Published:** 2025-05-09

**Authors:** Guogen Shan, Hui Li, Yahui Zhang, Guoqiao Wang, Charles Bernick

**Affiliations:** ^1^Department of Biostatistics, University of Florida, Gainesville, FL, United States; ^2^School of Smart Health, Chongqing Polytechnic University of Electronic Technology, Chongqing, China; ^3^School of Medicine, Washington University St Louis, St. Louis, MO, United States; ^4^Cleveland Clinic Lou Ruvo Center for Brain Health, Las Vegas, NV, United States

**Keywords:** Alzheimer’s disease, blood biomarkers, cognitive measures, NfL, p-tau181, surrogate endpoint

## Abstract

**Background:**

Blood biomarkers for Alzheimer’s disease (AD) can be utilized as surrogate endpoints to accelerate therapeutic development for this condition.

**Methods:**

We assessed the association between short-term changes in blood biomarkers and long-term declines in cognitive and brain structure volume measures using the ADNI database, with a focus on amyloid-*β* (A*β*)-positive participants. In our statistical models, the association was calculated after controlling for age, sex, and other possible confounding covariates. Additionally, outliers were removed before running the statistical models to ensure that the results were robust.

**Results:**

A trend association was found between changes in the levels of plasma neurofilament light (NfL) at 12 months and changes in the scores on the Mini-Mental State Examination (MMSE) at 24 months in Aβ-positive mild cognitive impairment (MCI) patients. For A*β-*positive dementia patients, a trend association was observed between changes in plasma p-tau181 levels and changes in whole brain and middle temporal volume.

**Conclusion:**

Increased plasma levels of NfL or p-tau181 blood biomarkers were found to be associated with reduced brain volumes and/or declined cognitive outcomes, suggesting that these blood biomarkers may have a predictive role in AD trials.

## 1 Background

Alzheimer’s disease (AD) is a complex neurodegenerative disorder characterized by a prolonged pre-clinical phase. During this phase, the accumulation of extracellular amyloid-*β* (A*β*) plaques and intraneuronal neurofibrillary tangles (NFTs) leads to brain damage (Ashton et al., 2021). Plasma tau phosphorylated (p-tau) protein is a major component of NFTs. Both A*β* and p-tau can be detected using positron emission tomography (PET), and they have a high accuracy in predicting AD (Mattsson et al., 2016; Hampel et al., 2018; Norgren et al., 2003). However, PET imaging comes with a high medical cost. There is growing evidence that inexpensive and easily available blood biomarkers can be used as potential tools for the early diagnosis and tracking the progression of AD (Ashton et al., 2021; Mattsson et al., 2019). These blood biomarkers include p-tau at threonine-181 (p-tau181) and the neurofilament light chain (NfL) (Chatterjee et al., 2023; Cummings et al., 2018; Zetterberg and Schott, 2019). Dementia patients had increased levels of p-tau181 compared to cognitively unimpaired (CN) individuals. This elevation is associated with elevated levels of brain A*β* plaques and tau NFTs (Mielke et al., 2018; Janelidze et al., 2020; Karikari et al., 2020; Shan et al., 2022; Lantero Rodriguez et al., 2020; Benussi et al., 2020; Chen et al., 2021). As a potential specific blood biomarker for AD, abnormal concentrations of plasma p-tau181 become evident in the preclinical stage of the disease. This biomarker differentiates AD dementia from other non-AD neurodegenerative disorders and helps predict the subsequent risk of developing AD dementia in cognitively unimpaired individuals and MCI patients (Janelidze et al., 2020; Cummings et al., 2021; Lantero Rodriguez et al., 2020; De Meyer et al., 2022; Norgren et al., 2003; Mattsson et al., 2017). Additionally, high levels of p-tau181 have been linked to a faster rate of decline in cognitive function, as measured by the Mini-Mental State Examination (MMSE), in individuals with AD and MCI and a shorter duration to show a clinically significant functional decline (Tropea et al., 2023).

Elevated baseline levels of NfL have been linked to poor cognitive performance and accelerated disease progression, with the rate of change correlating with cognitive changes in patients with AD (Mielke et al., 2019; Mattsson et al., 2019; Raket et al., 2020). Longitudinal changes in NfL were found to be associated with longitudinal cognitive decline in patients with cognitive impairment (AD and MCI combined) (Moscoso et al., 2021). In a pilot randomized controlled trial designed to assess the effects of aerobic exercise in AD (the FIT-AD trial) (Li et al., 2021), baseline plasma NfL levels and the changes observed after 3 months were associated with changes in cognition and the ability to perform activities of daily living.

Establishing an association between short-term changes in blood biomarker and long-term changes in cognitive measure may help support the use of blood biomarkers as predictors of progression and even as surrogate endpoints in AD research. This concept is similar to using response endpoints (e.g., whether a patient has responded to treatment) in early-phase cancer trials as surrogate endpoints for survival in later-phase trials (Kemp and Prasad, 2017; Shan et al., 2012; Shan et al., 2016; Shan, 2021; Shan, 2023). Blood biomarker data are easy to obtain and can be measured quickly as compared to cognitive outcomes in phase 3 AD trials. The primary outcome in the phase 3 lecanemab trial was the change in the Clinical Dementia Rating Scale Sum of Boxes (CDR-SB) score at 18 months from baseline (van Dyck et al., 2023). In phase 2 AD trials, the follow-up time (e.g., 12 months) may be shorter than in phase 3 trials (Swanson et al., 2021); however, it is still significantly longer than the time required to assess blood biomarkers. Blood biomarkers can be measured in an inexpensive and timely manner. The objective of this study is to assess the association between short-term blood biomarker data and long-term cognitive and brain structure volume measures, by using data from the Alzheimer’s Disease Neuroimaging Initiative (ADNI) study (Weiner et al., 2012). The interaction analysis was performed to study the association between participants with CN, MCI, and dementia.

## 2 Methods

### 2.1 Data sets

We utilized data from the ADNI study to assess the association between short-term blood biomarkers and long-term cognitive and brain structure volume measures (Weiner et al., 2015; Jagust et al., 2010). Data were downloaded on 12 June 2023 for analysis. Patients with p-tau181 and NfL data were included in the study, resulting in a total of 1,179 patients. To ensure robust results, participants with outliers were removed from the study. Outliers were defined as p-tau181 levels above 90, NfL levels above 199, or changes in p-tau181 and NfL levels at 12 months from baseline above 50 or below −50. The values of these outlier cutpoints were based on the findings from the article by Moscoso et al. (2021), with some modifications. The NfL outlier cutpoint was based on the maximum value of NfL found in their published results, and the cutpoint for p-tau181 was set slightly more conservatively compared to the range mentioned in their results (90 vs. 72) (Moscoso et al., 2021). The annual change cutpoint selected for this analysis was conservative. After removing these outliers, 1,155 participants were retained. As we focused on the cohort with positive amyloid levels, we had 558 Aβ-positive participants. Among them, there were 117 CN individuals, 284 MCI patients, and 157 dementia patients. In the ADNI study, participants were classified at each visit as either CN, MCI, or dementia. They were mostly from the ADNI-2 cohort (73%), followed by the ADNI-1 cohort (17%) and the ADNI-go cohort (10%). In the ADNI study, participants are assessed at baseline, 6, 12, 18, and 24 months in the first 2 years. After that, they have annual visits starting in the third year.

A*β* status was determined by using florbetapir (18F-AV-45) with a threshold value of 1.11 for the global standard uptake value ratio (SUVR) (Landau et al., 2015). The study population consists of A*β-*positive patients, who are often the main study population in AD trials for disease-modifying therapies (DMTs).

### 2.2 Cognitive measures

Cognitive data are collected at every in-clinic visit, and many cognitive assessments and questionnaires are administered by clinicians. In some cognitive assessments, a study partner is required to answer the related questions. More details can be found at: https://adni.loni.usc.edu/data-samples/adni-data/clinical-assessments/.

We included the most commonly used cognitive measures in clinical trials: CDR-SB, MMSE, and 13-item Alzheimer’s Disease Assessment Scale-Cognitive Subscale (ADAS-Cog version 13) (ADAS-Cog13) (Schultz-Larsen et al., 2007; Wang et al., 2016). These measures can be obtained from the combined ADNI file (ADNIMERGE.csv). A higher score of CDR-SB or ADAS-Cog13 indicates a more severe stage of the disease, while the MMSE score shows an inverse relationship. To maintain consistency with the other two cognitive measures, we used the revised MMSE score as 30-MMSE in our data analysis. For all the three cognitive measures, the association between their changes in p-tau181 or NfL levels should be positive.

### 2.3 Brain structure volume measures

ADNI-1 images were obtained using 1.5 T scanners, with one-fourth of the participants also scanned on 3 T. In the late ADNI phases (e.g., ADNI-Go and ADNI-2), all imaging data were processed exclusively on 3 T scanners. As MRI vendors provide online corrections, ADNI utilized these vendor-provided pre-processing techniques xrather than conducting its pre-processing. The volumes of the region of interests (ROIs) were reconstructed using FreeSurfer software; more details can be found at https://adni.loni.usc.edu/data-samples/adni-data/neuroimaging/mri/.

From the combined ADNI file, five AD-related brain regional volumes were included in the analysis: the middle temporal, hippocampal, ventricular, entorhinal, and whole brain volumes. These five brain regions were selected due to the known relationship between the volume of these areas and disease progression (Pini et al., 2016). During the development of AD pathology, atrophy in the entorhinal and hippocampal regions occurred very early, making them well-established and validated core biomarkers (Pini et al., 2016). Atrophy affects the whole brain and extends to the cortex along the temporal–parietal–frontal axis. Meanwhile, the spaces filled with fluid made the ventricles larger. Therefore, these five AD-related brain regional volumes were selected for analysis.

### 2.4 Blood biomarker measures

Plasma p-tau181 samples were obtained from the Clinical Neurochemistry Laboratory, University of Gothenburg, Sweden on 8 June 2020 (Karikari et al., 2020), and plasma NfL samples were provided by the same group using the ultrasensitive Single-Molecule-Array (SIMOA) technology platform, for more details, visit: https://adni.loni.usc.edu/data-samples/adni-data/biofluid-biomarker/. Detailed information about the collection and preparation of plasma samples is aligned with best practice guidelines, as discussed previously (Zicha et al., 2023).

Other covariates obtained from the combined ADNI file included age, sex, years of education, and counts of the apolipoprotein E (ApoE)-*ε*4 gene.

### 2.5 Statistical analysis

In our study cohort, which consisted of 558 Aβ-positive participants from the ADNI study, we calculated the mean and standard deviation (SD) for continuous variables and the proportions for categorical variables across each group (CN, MCI, and dementia). The Kruskal–Wallis test was used to compare differences among groups for continuous outcomes, whereas Fisher’s exact test was used for categorical data (Shan, 2016; Shan et al., 2020). Data from the first visit with NfL or p-tau181 were used to calculate the characteristics of the study cohort. Since NfL and p-tau181 data were skewed, a log_10_ transformation was applied to the plasma biomarker data in the statistical models (Snellman et al., 2023).

The short-term blood biomarker data reflect the changes observed at 12 months from baseline, while long-term changes in cognitive or brain structure measures were assessed at 24 months or longer from baseline. Their association was analyzed using multiple linear regression models after controlling for their baseline measures, age, sex, years of education, and counts of ApoE-*ε*4. We calculated the raw *p*-values and the adjusted *p*-values by using the Benjamini-Hochberg approach (Benjamini and Hochberg, 1995). We used SAS (version 9.4) for data analysis (Shan et al., 2018). In the following section, we performed the analysis with the long-term change as the outcome, and we examined the interaction between short-term blood biomarker changes and cognitive status. In this analysis, data from all groups were included in the model. From the fitted model, we reported the association for each cognitive status with the standardized coefficient estimate (b) and its standard error (SE). The 95% confidence interval based on the standardized parameter can be calculated as follows: standardized coefficient estimate *±* 1.96 *×* SE. We reported the raw *p*-values in the next section and the adjusted *p*-values after considering multiple comparison corrections in the tables.

## 3 Results

Table 1 summarizes the participants’ characteristics. In this cohort, more female participants (64.1%) were enrolled in the CN group (64.1%) as compared to those in MCI (43.0%) and dementia (45.2%) groups. This difference is statistically significant with a *p*-value of 0.0004, as determined by Fisher’s exact test for proportions (Shan et al., 2016; Shan, 2015). In this cohort, the majority of participants were white individuals (over 90% in each group) (Weber et al., 2021) and non-Hispanic population (over 94% in each group), which is the focus of the current ADNI-4 to increase participant diversity in the future (Weiner et al., 2023). The ApoE-*ε*4 positivity rate is expected to be much higher in MCI and dementia patients than in healthy controls. Both blood biomarkers reached their highest levels in dementia patients, followed by MCI and CN individuals. A similar trend was observed for the three cognitive measures and the five AD-related brain regional volumes. Of the 558 participants with baseline blood biomarker data, 367, 221, and 130 had available data at the 24-, 26-, and 48-month follow-ups, respectively.

**Table 1:** Characteristics of A*β-*positive participants from the ADNI study having NfL and p-tau181 measures.

Measure	Overall	CN	MCI	Dementia	*p*-value
Sample size	*n* = 558	*n* = 117	*n* = 284	*n* = 157	
Age, years	73.6 (7.0)	74.8 (5.7)	73.3 (6.9)	73.5 (7.9)	0.2619
Education, years	15.9 (2.8)	16.2 (2.8)	15.9 (2.9)	15.8 (2.7)	0.5274
Female	268 (48.0%)	75 (64.1%)	122 (43.0%)	71 (45.2%)	0.0004
Non-hispanic	536 (99.3%)	112 (95.7%)	276 (97.2%)	148 (94.3%)	0.3297
White	521 (93.4%)	107 (91.5%)	269 (94.7%)	145 (92.4%)	0.2940
ApoE e4 counts					*<*0.0001
0 count	202 (36.20%)	64 (54.70%)	100 (35.21%)	38 (24.20%)	
1 count	272 (48.75%)	49 (41.88%)	142 (50.00%)	81 (51.59%)	
2 counts	84 (15.05%)	4 (3.42%)	42 (14.79%)	38 (24.20%)	
NfL, pg./mL	43.4 (20.2)	39.1 (15.5)	42.7 (21.7)	47.9 (19.9)	0.0001
p-tau181, pg./mL	21.5 (9.9)	17.0 (7.5)	21.5 (10.8)	24.8 (8.3)	*<*0.0001
CDR-SB	2.3 (2.3)	0.1 (0.3)	1.6 (1.1)	5.0 (2.3)	*<*0.0001
ADAS-Cog13	19.4 (10.9)	9.9 (4.4)	16.6 (6.9)	31.9 (9.3)	*<*0.0001
MMSE	26.5 (3.3)	28.9 (1.2)	27.6 (1.8)	22.6 (3.0)	*<*0.0001
Hippocampal, cm^3^	7.0 (1.1)	7.2 (0.9)	6.8 (1.0)	5.8 (1.0)	*<*0.0001
Middle temporal, cm^3^	19.3 (3.1)	20.2 (2.8)	19.9 (2.8)	17.3 (3.1)	*<*0.0001
Entorhinal, cm^3^	3.4 (0.7)	3.7 (0.5)	3.5 (0.7)	2.8 (0.7)	*<*0.0001
Ventricular, cm^3^	42.1 (23.2)	37.2 (20.8)	39.6 (22.7)	50.3 (23.9)	*<*0.0001
Whole brain, cm^3^	1027.6 (110.4)	1022.7 (105.4)	1047.2 (103.3)	993.2 (118.9)	*<*0.0001

In Table 2, we presented the association between short-term log(NfL) changes and long-term cognitive and brain structure volume changes, after controlling for age, sex, years of education, counts of ApoE-*ε*4, baseline log(NfL), and baseline of the measure of interest. For A*β-*positive MCI patients, the change in log(NfL) at 12 months from baseline (specifically a 2.0 yearly increase in the NfL measure) was associated with the change in the MMSE score in 24 months (b = 2.88, *p* = 0.030), where b is the standardized coefficient estimate, and p is the raw p-value. For Aβ-positive dementia patients, the short-term change in log(NfL) was estimated as a 6.3 yearly increase in the NfL measure. We noticed a trend in the association between the short-term change in log(NfL) and the change in the ADAS-Cog13 score (*b* = 6.26, *p* = 0.057). The interaction analysis revealed a subgroup difference in the association between the short-term change in log(NfL) and middle temporal volume (*p* = 0.017), indicating that the dementia group had a faster rate of disease progression. However, we found no further differences in the interaction analysis.

**Table 2:** For Amyloid-*β-*positive participants, association between log(NfL) change in 12 months and cognitive and volume measures change in 24 months after controlling for age, sex, education, ApoE-e4 counts, baseline log(NfL), and baseline of the considered measure.

	A*β-*positive CN		A*β-*positive MCI		A*β-*positive Dementia	
	b (SE)	p (p-adj)	b (SE)	p (p-adj)	b (SE)	p (p-adj)
Cognitive measures						
CDR-SB	0.32 (0.77)	0.679 (0.905)	1.06 (0.77)	0.169 (0.337)	0.23 (0.82)	0.783 (0.783)
ADAS-cog-13	0.03 (3.06)	0.993 (0.993)	5.50 (3.07)	0.074 (0.198)	6.26 (3.28)	0.057 (0.180)
Reversed MMSE	0.97 (1.31)	0.459 (0.746)	2.88 (1.32)	0.030 (0.198)	−0.45 (1.40)	0.746 (0.783)
Brain structure volume measures						
Middle temporal, dm^3^	1.02 (0.45)	0.023 (0.184)	0.36 (0.45)	0.420 (0.634)	−1.02 (0.52)	0.051 (0.180)
Hippocampal, dm^3^	0.24 (0.20)	0.223 (0.746)	−0.14 (0.20)	0.476 (0.634)	−0.19 (0.22)	0.393 (0.523)
Ventricular, dm^3^	1.22 (1.68)	0.466 (0.746)	3.04 (1.69)	0.074 (0.198)	1.87 (2.18)	0.392 (0.523)
Entorhinal, dm^3^	0.01 (0.18)	0.946 (0.993)	0.01 (0.18)	0.937 (0.937)	0.40 (0.22)	0.068 (0.180)
Whole brain, dm^3^	11.26 (11.85)	0.343 (0.746)	−2.84 (11.77)	0.810 (0.925)	−19.23 (13.87)	0.167 (0.335)

*p*, *p*-value; *p*-adj, *p*-value after the multiple correction by using the Benjamini-Hochberg approach. The value *b* is the standardized coefficient estimate. Reversed MMSE equals to 30 - MMSE original score.

**Figure 1:**
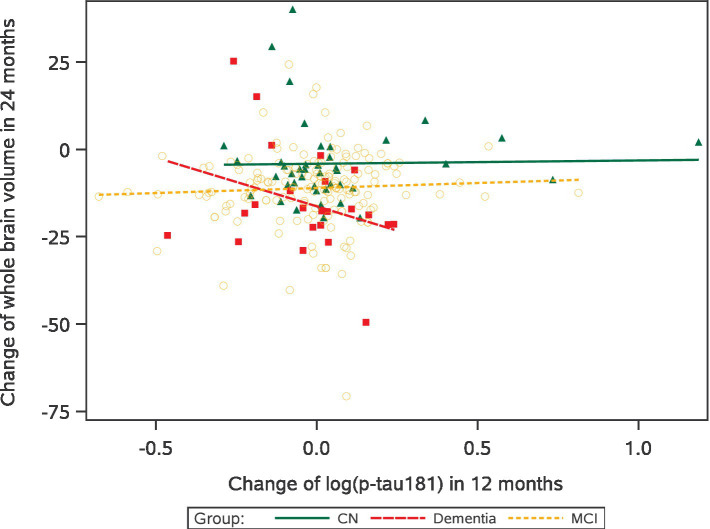
Association between change of whole brain volume (dm^3^) at 24 months and changes in log(p-tau181) at 12 months for amyloid-*β-*positive patients.

Table 3 shows the association between the short-term log(p-tau181) change at 12 months from baseline and the long-term change in cognitive or brain structure volume measures at 24 months from baseline for A*β-*positive participants. We found a significant association between entorhinal and log(p-tau181) changes in dementia patients (*b* = 0.58, *p* = 0.003), but the direction of the association was not as expected due to small sample sizes. In dementia patients, an increase in p-tau181 levels (2.4 yearly increase of the p-tau181 measure) was associated with a decrease in whole brain volume measure (b = −23.01, *p* = 0.055), see Figure 1. The interaction analysis does not indicate any significant subgroup differences.

**Table 3:** For Amyloid *β-*positive participants, association between log(p-tau181) change in 12 months and cognitive and volume measures change in 24 months after controlling for age, sex, education, ApoE-e4 counts, baseline log(p-tau181), and baseline of the considered measure.

	A*β-*positive CN		A*β-*positive MCI		A*β-*positive Dementia	
	b (SE)	p (p-adj)	b (SE)	p (p-adj)	b (SE)	p (p-adj)
Cognitive measures						
CDR-SB	0.63 (0.84)	0.456 (0.911)	1.40 (0.82)	0.087 (0.695)	0.50 (0.78)	0.524 (0.927)
ADAS-cog-13	1.11 (3.34)	0.741 (0.911)	1.64 (3.29)	0.619 (0.933)	1.04 (3.06)	0.734 (0.927)
Reversed MMSE	3.27 (1.40)	0.020 (0.160)	0.12 (1.37)	0.933 (0.933)	0.46 (1.28)	0.720 (0.927)
Brain structure volume measures						
Middle temporal, dm^3^	0.12 (0.51)	0.808 (0.911)	0.18 (0.50)	0.713 (0.933)	−0.85 (0.48)	0.079 (0.210)
Hippocampal, dm^3^	0.06 (0.22)	0.774 (0.911)	0.07 (0.21)	0.751 (0.933)	0.02 (0.20)	0.927 (0.927)
Ventricular, dm^3^	−0.39 (1.91)	0.838 (0.911)	−1.09 (1.86)	0.558 (0.933)	−0.17 (1.73)	0.923 (0.927)
Entorhinal, dm^3^	−0.14 (0.20)	0.504 (0.911)	0.10 (0.20)	0.622 (0.933)	0.58 (0.19)	0.003 (0.022)
Whole brain, dm^3^	−1.46 (13.03)	0.911 (0.911)	2.08 (12.70)	0.870 (0.933)	−23.01 (11.90)	0.055 (0.210)

*p*: *p*-value; *p*-adj: *p*-value after the multiple correction by using the Benjamini-Hochberg approach. The value *b* is the standardized coefficient estimate. Reversed MMSE equals to 30 - MMSE original score.

In addition to the association between the short-term change in blood biomarkers and the 24-month change in cognitive and brain structure volume measures, we studied the long-term change in longer follow-up visits in cognitive and brain structure volume measures. The change in the whole brain volume at 36 months from baseline was found to be associated with 12-month changes in both log(NfL) (*p* = 0.044) and log(p-tau181) (*p* = 0.031) in the dementia group. These findings suggest an association between the short-term blood biomarker and cognitive and brain volume measures at follow-up visits. The interaction analysis revealed that there might be differences between groups regarding the association between the change in two blood biomarkers at 36 months and whole brain volume measure.

## 4 Discussion

Recently, the Food and Drug Administration (FDA) granted breakthrough device designation to the Elecsys amyloid plasma panel (Pais et al., 2023; Cummings and Kinney, 2022). This blood biomarker test provides results for the p-tau181 protein assay and the APOE *ε*4 assay in human plasma. Blood biomarker tests can facilitate early diagnosis and enable timely intervention through appropriate care plans for patients and their caregivers. Additionally, the results from these tests can help reduce the time and cost of research studies on AD.

We expanded the current literature on assessing the association between short-term changes in plasma levels of p-tau181 and NfL and long-term cognitive and brain structure volume changes by using the ADNI data, with the focus on A*β-*positive patients who are the primary study population in AD drug development trials. The interesting findings were observed among the dementia group for volume measures: reduction in both middle temporal volume and whole brain volume among dementia patients. However, due to the small sample size and the limited number of significant findings after applying multiple corrections, additional data are required to confirm the findings of this study.

We found that the change in NfL and p-tau181 was associated with the change in middle temporal volume, with the raw *p*-value being very close to the nominal level. We may compare their association by using the estimated effect size. In general, a measure with a smaller *p*-value has a larger effect size. Our findings revealed that p-tau181 showed a slightly lower *p*-value than NfL, but their difference was small. A larger study is needed to confirm the use of p-tau181 in clinical trials.

According to the randomized FIT-AD trial (Li et al., 2021), changes in plasma NfL at 3 months from baseline were associated with decline in activities of daily living (ADL) at 6 months and 12 months from 3 months. The short-term change in NfL was also found to be associated with the change in memory at 6 months from 3 months. The abovementioned study focused on assessing the effect of short-term NfL change (3 months from baseline) on the future cognitive changes (with measures at 3 months as baseline). Meanwhile, we focused on the short-term change in blood biomarkers and the long-term change in cognitive and brain structure volume measures from baseline. In addition, we included findings related to the long-term change in brain structure volume measures in this article, which revealed some new insights.

To use blood biomarker data as surrogate endpoints in clinical trials, several operational criteria should be satisfied in AD research, including the association between surrogate endpoints and cognitive change, as well as the treatment effects on reducing blood biomarkers (Planche and Villain, 2021; Ashton et al., 2024; Cummings et al., 2018). Blood biomarker data are easier to collect than PET data. Baseline NfL levels were associated with the change in global mental status among white individuals in the Healthy Aging in Neighborhoods of Diversity across the Life Span (HANDLS) study (Beydoun et al., 2021). The change in NfL was found to be associated with the change in cognitive outcome measures (Mattsson et al., 2019).

NfL levels have been found to be elevated in multiple neurodegenerative disorders, including AD. It is an axonal component found primarily in large-caliber myelinated subcortical fibers, is one of the most widely studied fluid biomarkers, and is considered to be released during axonal injury. Thus, higher levels of NfL may reflect the presence of a more vigorous neurodegenerative process that is likely to progress. Elevated plasma p-Tau181 levels indicate the presence of brain amyloid plaque. However, the absolute levels of amyloid plaque do not tightly correlate with symptom severity and may be less reliable as predictors of the rate of cognitive decline. The field of biomarker development is evolving rapidly, and it is possible that a different combination of analytes measured over a short period may perform better than those included in this study.

NfL may be used to improve the prediction of AD dementia and vascular dementia (Wang et al., 2024). In addition, NfL has been suggested as a biomarker to monitor treatment response in relapsing–remitting multiple sclerosis (MS) (Kuhle et al., 2019). In the FREEDOMS trial, the NfL measure in the treatment fingolimod group decreased by more than 35% after 6 months from baseline. NfL levels could be affected by the drugs a patient is taking before and during the study. In addition, these levels could be affected by other factors, such as age and hypertension (Fitzgerald et al., 2022).

## 5 Limitations

In the ADNI database, we have the blood biomarker data collected during yearly visits, except for ADNI-1 participants who were followed at the 6-month mark. To determine the earliest time that shows a strong association between the short-term blood biomarker data and the long-term cognitive measures, a study involving more frequent visits (e.g., baseline, 3 months, 6 months, 9 months, and 12 months) would be necessary. In the FIT-AD trial (Li et al., 2021), blood samples were collected at baseline, 3 months, and 6 months to assess the association between the change in short-term blood biomarkers at 3 months from baseline and the long-term cognitive and functional changes at 6 or 12 months from 3 months. In their report, they mentioned the influence of outliers in the statistical analysis (Li et al., 2021). To ensure robust results, we removed patients with outliers in this article.

In this article, we focused on patients with positive A*β* for our data analysis. This population is commonly included in AD trials to assess the effectiveness of new AD drugs (Kuhle et al., 2019; van Dyck et al., 2023; Fitzgerald et al., 2022). To compare the MCI-to-AD conversion rate, we would include both Aβ-positive and Aβ-negative MCI patients in the model to identify different factors that affect the conversion time (Sims et al., 2023). In addition to raw *p*-values, we also reported adjusted p-values as the analysis was compared within each cognitive status. After multiple comparison corrections, some significant findings were no longer significant. The raw p-value is still informative for future clinical trials.

Several studies suggested that the A*β* in plasma may not be as accurate as the CSF or PET imaging biomarkers due to the variation in plasma (Zhang et al., 2024). The concordance between CSF or PET imaging biomarkers and plasma biomarkers were estimated to be between 66 and 95%, based on data from two studies: ADNI and BioFINDER-2 (Shan et al., 2021). Both CSF and PET biomarkers are often used in clinical settings. Compared to CSF or PET biomarkers, plasma biomarkers are easy to obtain at a low cost. Plasma biomarkers are critical in early AD clinical trials (Leuzy et al., 2022).

## 6 Conclusion

We used data from the ADNI study to examine the association between the short-term blood biomarker change at 12 months from baseline and the long-term change in cognitive and brain volume measures. One of the goals of this project was to investigate the possibility of using blood biomarkers as surrogate endpoints or predictors of change in AD research (Ossenkoppele et al., 2021; Oosthoek et al., 2024; van Dyck et al., 2023; Shan et al., 2024; Bernick et al., 2018). Increased plasma levels of NfL or p-tau181 blood biomarkers were found to be associated with reduced brain volumes and/or declined cognitive outcomes, suggesting that these biomarkers may have a predictive role or as a surrogate endpoint in AD trials.

## Data Availability

Publicly available datasets were analyzed in this study. This data can be found here: adni website.
